# The cholinergic anti-inflammatory pathway alleviates acute lung injury

**DOI:** 10.1186/s10020-020-00184-0

**Published:** 2020-06-29

**Authors:** Ulf Andersson

**Affiliations:** Department of Women’s and Children’s Health, Karolinska Institutet at Karolinska University Hospital, Tomtebodavägen 18A, 17176 Stockholm, Sweden

**Keywords:** Pneumonia, ARDS, ALI, HMGB1, RAGE, TLR4, GTS-21, Nicotine, Pathogenesis, Hyperoxia, COVID-19, Vagus nerve stimulation

## Abstract

The ubiquiotous nuclear protein HMGB1 is extracellularly released by dying cells or activated innate immunity cells to promote inflammation. Extracellular HMGB1 plays a prominent role in the pathogenesis of acute lung injury of infectious as well as sterile origin including hyperoxia. Excessive amounts of systemic HMGB1 and HMGB1-partner molecule complexes can be retained in the pulmonary circulation indicated by a substantial reduction of HMGB1 plasma levels in arterial versus venous blood. The cholinergic antiinflammatory mechanism ameliorates pulmonary inflammation by inhibiting HMGB1 release and HMGB1 receptor expression. This comprehension was recently reinforced by results reported in Molecular Medicine by Sitapara and coworkers demonstrating that administration of an α7 nicotinic acetylcholine receptor agonist attenuated hyperoxia-induced acute inflammatory lung injury by alleviating the accumulation of HMGB1 in the airways and the circulation. Activating the cholinergic antiinflammatory path might be considered to alleviate severe COVID-19 with or without concurrent oxygen-induced lung injury.

## Background

Excessive pulmonary inflammation causes acute lung injury (ALI) and acute respiratory distress syndrome (ARDS), two major problems with an unmet clinical need to control (Matthay et al. 2012). Several disorders precipitate ALI and ARDS including bacterial and viral pneumonia, sepsis, aspiration of gastric contents, and major trauma. The outcome has gradually been improved by lung-protective ventilation minimizing barotrauma and oxygen toxicity and fluid-conservative management to reduce vascular pressure contributing to pulmonary proteinaceous edema in the setting of increased lung vascular permeability. The central pathophysiology leading to impaired gas exchange is caused by dysregulated inflammation with altered permeability of alveolar endothelial and epithelial barriers, inappropriate accumulation and activity of leukocytes and platelets and uncontrolled coagulation. However, these insights have not yet generated specific therapies that reduce morbidity and mortality in humans. It is conceivable that the release of damage-associated molecular pattern molecules (DAMPs) from dying cells and by activated innate immunity cells in the lungs exerts a central pathogenic mechanism. Identification and therapeutic targeting of such key molecules might offer novel prospects to improve outcome.

### HMGB1

One such intriguing candidate molecule is high mobility group box 1 protein (HMGB1). It is one of the most extensively studied DAMPs, present in high extracellular quantities and involved in the pathogenesis of many inflammatory diseases of infectious or sterile origin (Kang et al. 2014). It is a ubiquitous 25 kDa chromatin-binding protein present in all cells and the molecule is 99% identical in mammals. HMGB1 is passively extracellularly released as a prototypical DAMP from dying cells (except from apoptotic cells) or secreted by stressed or activated cells in any tissue. HMGB1 initiates inflammation using two separate receptor systems, which are TLR4 and the receptor for glycated endproducts (RAGE). Disulfide-HMGB1 triggers TLR4 receptors activating pro-inflammatory cytokine release. Extracellular HMGB1 may in addition form complexes with extracellular molecules including DNA, RNA and other DAMP or pathogen-associated molecular pattern (PAMP) molecules. These complexes are endocytosed via RAGE, constitutively expressed at high levels in the lungs *only (*Bierhaus et al. 2005*)*, and transported to the cellular endolysosomal system, which is disrupted by HMGB1 at high concentrations in the acidic intralysosomal environment (Deng et al. 2018). Danger molecules may thus get access to cytosolic proinflammatory receptors initiating inflammasome activation. The extracellular DAMPs and PAMPs would not reach their cognate cytosolic receptors without the HMGB1-assisted transport. It is plausible that these complexes are specifically removed in the lungs indicated by a 40% reduction of HMGB1 plasma levels in arterial versus venous blood (Ottestad et al. 2019). The abundant pulmonary RAGE expression enables endocytosis of danger molecules to get destructed in the lysosomes at physiological HMGB1 levels, but causing detrimental inflammasome activation at high levels. Another captivating observation connecting HMGB1 directly to lung biology is based on a recent publication reporting hypoxia-induced apoptosis in pulmonary endothelial cells from female mice but necrosis in cells from male mice (Zemskova et al. 2020). Necrosis mediates massive extracellular HMGB1 release, while apoptosis does not.

A global lack of clinically approved HMGB1-specific antagonists has so far precluded studies in patients with HMGB1-dependent inflammatory diseases. However, the HMGB1-neutralization strategy has been extensively studied with successful results in many preclinical models of inflammatory disorders including severe pulmonary inflammation. Systemic or local administration of HMGB1-specific monoclonal antibodies mediates beneficial therapeutic results in preclinical models of acute lung injury caused by bacterial infection, influenza- and adenoviruses, trauma-induced systemic inflammatory response syndrome (SIRS), hypoxia, hyperoxia, and barotrauma inflicted by mechanical ventilation (Andersson et al. 2020).

### Acute lung injury controlled by the cholinergic system

**Fig. 1:**
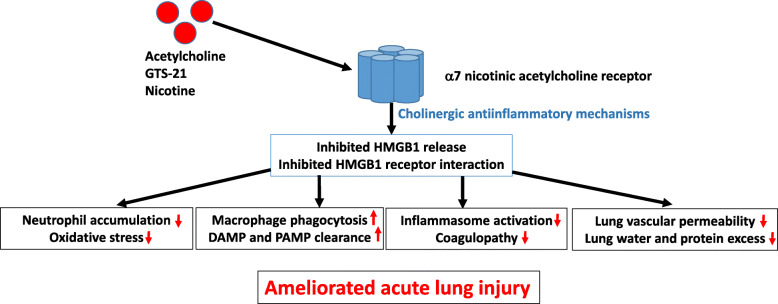
The cholinergic antiinflammatory pathway alleviates acute lung injury. Agonists to α7 nicotinic acetylcholine receptors reduce release of extracellular HMGB1 and expression of the two main HMGB1 receptors RAGE and TLR4. The inhibition of HMGB1-driven inflammation ameliorates the development of acute lung injury and ARDS by constraining certain and supporting other cellular functions outlined in the figure

Iatrogenic hyperoxia is one of many sterile causes of acute lung injury associated with massive local extracellular HMGB1 release and neutrophil accumulation. Intratracheal administration of HMGB1 induces a significant inflammatory response characterized by the infiltration of neutrophils playing a pivotal role in pulmonary inflammation (Entezari et al. 2014). Mechanical ventilation with supraphysiological concentrations of oxygen has been shown to compromise the ability of alveolar macrophages to clear bacteria predisposing to pneumonia generation. Preclinical studies indicate a pathogenic role for HMGB1 in this hyperoxia-induced impairment (Patel et al. 2013). This notion was further supported in another experimental study demonstrating that GTS-21 treatment effectively improved bacterial clearance and reduced acute lung injury by enhancing macrophage function via inhibiting the release of HMGB1 from macrophages (Sitapara et al. 2014) (Fig.1).

Prolonged exposure to hyperoxia is associated with oxygen toxicity, due to the excessive production of reactive oxygen species, which may lead to oxidative stress-mediated hyperoxia-induced acute lung injury (HALI) (Kallet and Matthay 2013). The increased infiltration of ROS-releasing neutrophils causes further oxidative stress-induced cell damage and lung injury. A recent study published in Molecular Medicine by Sitapara et al reported about the therapeutic effect of GTS-21, an α7 nicotinic acetylcholine receptor agonist (α7 nAChR), attenuating HALI in a mouse model (Mol Med 2020; in press). The authors used ex vivo bronchoalveolar lavage (BAL) fluid and lung tissue samples from HALI mice treated with GTS-21 to determine the levels of HMGB1 and tissue inflammation and damage. Furthermore, flow cytometry analysis was performed on BAL fluid samples to demonstrate the effect of GTS-21 on immune cell infiltration. The key finding of this work was that systemic GTS-21 administration ameliorated HALI by alleviating the accumulation of HMGB1 and neutrophils in the airways and in the circulation. Furthermore, GTS-21 therapy also improved macrophage phagocytosis needed for optimal clearance of invading pathogens and dying neutrophils. These are observations of potentially great clinical and therapeutic interest provided that the treatment would also work in patients receiving oxygen therapy for severe pneumonia.

What was then the rational for treating acute lung injury with an α7nAChR agonist? The immune system is like all other organs controlled by the central nervous system. The vagus nerve-mediated inflammatory reflex with its efferent arm the cholinergic antiinflammatory pathway is so far the most extensively studied example of an immunoregulatory neural circuit (Chavan et al. 2017). Acetylcholine, nicotine, and α7nAChR-specific agonists all act via α7 nAChR expressed on alveolar macrophages and lung epithelial cells to downregulate pulmonary inflammation. Specific α7nAChR agonist therapy in a preclinical study of acid-induced acute lung injury decreased excess lung water, lung vascular permeability, and reduced protein concentration in the bronchoalveolar lavage fluid (Fig. 1). Alpha7nAChR gene-deficient mice expressed a 2-fold increase in excess lung water and lung vascular permeability in the same model (Su et al. 2007). Activation of the α7nAChR prevents activation of the NF-κB pathway and inhibits HMGB1 secretion from cultured human macrophages (Wang et al. 2004). GST-21 as well as vagus nerve stimulation treatments attenuated serum HMGB1 levels and improved survival in experimental models of sepsis (Pavlov et al. 2007; Huston et al. 2007). The cholinergic anti-inflammatory pathway not only downregulates HMGB1 release but also inhibits HMGB1 receptor-mediated activities. RAGE-mediated endocytosis of HMGB1 complexes with other proinflammatory molecules has been demonstrated to be constrained in cultured macrophages by acetylcholine as well as GTS-21 (Yang et al. 2019). The inhibited endocytosis led to reduced release of proinflammatory cytokines and pyroptosis. Furthermore, acetylcholine signaling via α7nAChR has also been reported to protect against LPS-induced acute lung injury by inhibiting the TLR4/MyD88/NF-κB pathway (Zi et al. 2019) (Fig.1) .

Acetylcholine-mediated amelioration of inflammation may in addition be accomplished by electrical stimulation of the left vagus nerve (Chavan et al. 2017). Surgical implantation of vagus nerve pacemakers mediates highly beneficial therapeutical results in patients with polyarthritis and inflammatory bowel disease (Koopman et al. 2016; Bonaz et al. 2016). The need for surgery can be circumvented by external transauricular vagus nerve stimulation using an external pulse generator, which is an inexpensive device meant for personal use. Furthermore, auricular transcutaneous vagus nerve stimulation reduced systemic HMGB1 levels and improved survival in an experimental sepsis model (Huston et al. 2007). Alpha 7nAchR-signaling inhibits inflammasome activation by preventing release of mitochondrial DNA (Lu et al. 2014). Increased extracellular ATP levels enable acetylcholine to translocate into the cytoplasm of innate immunity cells to bind and activate α7nAchR abundantly expressed on the surface of mitochondria. Inhibited inflammasome activities reduce the release of HMGB1, IL-1α, IL-1β, and IL-18 and diminish the risk of coagulopathy (Fig. 1).

## Conclusions

The need for prolonged mechanical ventilation using high oxygen concentration is a major problem connected to the care for COVID-19 patients. The discovery by Sitapara and colleagues that hyperoxia-induced acute lung injury was ameliorated via α7nAChR-agonist may offer important clinical progress if confirmed in future studies in patients. It should be noted that GTS-21 has been clinically tested in healthy adult volunteers and patients with schizophrenia demonstrating a favorable safety profile (Lewis et al. 2018). Furthermore, vagal stimulation by implanted or external electrical pulse generators has also been successfully studied in humans in inflammatory conditions with beneficial and safe outcomes.

## Data Availability

Not applicable.

## Abbreviations

ALI: Acute lung injury
ARDS: Acute respiratory distress syndrome
GTS-21: [3-(2,4 dimethoxy- benzylidene)-anabaseine dihydrochloride]
α7nAChR: Alpha 7 nicotinic acetylcholine receptor
BAL: Bronchoalveolar lavage
HALI: Hyperoxia induced acute lung injury
HMGB1: High mobility group box protein 1
Hyperoxia: Greater than 99% oxygen
DAMP: Damage-associated molecular pattern
PAMP: Pathogen-associated-molecular-pattern
COVID-19: Coronavirus disease 2019
LPS: Lipopolysaccharide
RAGE: Receptor for advanced glycation end-product
TLR4: Toll-like receptor 4
